# DeepCOMBI: explainable artificial intelligence for the analysis and discovery in genome-wide association studies

**DOI:** 10.1093/nargab/lqab065

**Published:** 2021-07-20

**Authors:** Bettina Mieth, Alexandre Rozier, Juan Antonio Rodriguez, Marina M C Höhne, Nico Görnitz, Klaus-Robert Müller

**Affiliations:** Machine Learning Group, Technische Universität Berlin, Berlin 10587, Germany; Max Planck School of Cognition, Stephanstrasse 1a, Leipzig 04103, Germany; Machine Learning Group, Technische Universität Berlin, Berlin 10587, Germany; RTE Réseau de Transport d’Electricité, Paris 92800, France; CNAG-CRG, Centre for Genomic Regulation (CRG), Barcelona Institute of Science and Technology (BIST), Barcelona 08003, Spain; Machine Learning Group, Technische Universität Berlin, Berlin 10587, Germany; Understandable Machine Intelligence Lab, Technische Universität Berlin, Berlin 10587, Germany; 123ai.de, Berlin 10319, Germany; Machine Learning Group, Technische Universität Berlin, Berlin 10587, Germany; Department of Artificial Intelligence, Korea University, Seoul 02841, Republic of Korea; Max Planck Institute for Informatics, Saarbrücken 66123, Germany

## Abstract

Deep learning has revolutionized data science in many fields by greatly improving prediction performances in comparison to conventional approaches. Recently, explainable artificial intelligence has emerged as an area of research that goes beyond pure prediction improvement by extracting knowledge from deep learning methodologies through the interpretation of their results. We investigate such explanations to explore the genetic architectures of phenotypes in genome-wide association studies. Instead of testing each position in the genome individually, the novel three-step algorithm, called DeepCOMBI, first trains a neural network for the classification of subjects into their respective phenotypes. Second, it explains the classifiers’ decisions by applying layer-wise relevance propagation as one example from the pool of explanation techniques. The resulting importance scores are eventually used to determine a subset of the most relevant locations for multiple hypothesis testing in the third step. The performance of DeepCOMBI in terms of power and precision is investigated on generated datasets and a 2007 study. Verification of the latter is achieved by validating all findings with independent studies published up until 2020. DeepCOMBI is shown to outperform ordinary raw *P*-value thresholding and other baseline methods. Two novel disease associations (rs10889923 for hypertension, rs4769283 for type 1 diabetes) were identified.

## INTRODUCTION

Genome-wide association studies (GWAS) investigate the phenotypic effects of small genetic variations called single-nucleotide polymorphism (SNPs). While some methods for the analysis of GWAS focus on phenotypic risk prediction based on the given genetic information (1–5), others try to explain these risk effects by highlighting which SNPs are having an effect on a given trait (6–10). This work aims at a combination of both of these goals and uses a deep learning-based prediction method in combination with statistical testing to identify SNPs associated with the phenotype under investigation.

Following developments in biotechnology, the first GWAS was published in 2002 (11–13). Several years later, a landmark study—the largest GWAS ever conducted at the time of its publication in 2007—was presented by the Wellcome Trust Case Control Consortium (WTCCC) (14) including 14 000 cases of seven common diseases and 3000 shared controls. Ever since then, sample sizes, rates of discovery and numbers of traits studied have been rising continuously (15). According to the GWAS catalog accessed on 15 September 2020, >4700 studies have investigated >3,500 phenotypes and identified >200,000 SNP phenotype associations with *P*-values below 1 × 10^–5^. Especially for common human diseases such as diabetes, autoimmune disorders or psychiatric illnesses, GWAS have provided valuable insight into the corresponding genetic inheritance processes (16). A few studies have included over 1 million subjects enabling the identification of SNPs with lower risks and frequencies (17,18).

However, the vast amount of available data on SNP phenotype associations still only accounts for a small fraction of heritability. The genetic architectures and variances of most traits and diseases remain largely unexplained. This effect, often referred to as ‘the missing heritability’, is assumed to—at least partially—be caused by the way GWAS datasets are traditionally analyzed (19,20). The classic approach—which we refer to as raw *P*-value thresholding (RPVT)—consists of carrying out a statistical association test to assign a *P*-value to each SNP under investigation and subsequently assessing its statistical significance via comparison to a predefined threshold ${t^*}$ (16). This standard approach to analyzing GWAS is therefore based on testing SNPs individually and in parallel, which intrinsically ignores any potential interactions (20,21) between or correlation structures among the set of SNPs under investigation (22,23). Studies fail to identify multi-locus effects by using the traditional RPVT approaches and a large amount of potentially available information is lost (24). Only very few diseases rely on single genetic defects with large effects. Most complex diseases are caused by epistatic interactions of multiple genetic factors with small effect sizes, which are further influenced by correlation structures due to both population genetics and biological relations (15). Brute force multivariate approaches to identify such dependencies are oftentimes computationally too expensive for large GWAS datasets and are limited by low statistical power due to excessive multiple testing. A few attempts have been made to identify genetic interactions, but most of them were not able to find strong, statistically significant associations (21,25–27).

To overcome these limitations of traditional approaches and following the rise of machine learning in data science and an increasing amount of available large-scale GWAS datasets, a number of methods have been proposed to introduce machine learning tools for the analysis of such studies. Linear approaches such as multivariate logistic regression and sparse penalized methods including Lasso have been applied to GWAS datasets. In general, penalized models achieve better performances than nonpenalized methods (4,28–30). Some of the top-performing models combine statistical testing and machine learning for the identification of SNP disease associations (6,7,31). While most of these methods do not provide validation on real data comparing to the GWAS database, very few provide a full evaluation of identified genetic variants in terms of comparison to previously published GWAS. Other proposed nonlinear models, such as random forests, gradient boosted trees and Bayesian models (4,28,32,33), investigate interactions and correlations in the genetic architecture of traits but were mostly found to be outperformed by linear penalized methods (4,25,28,33).

To harness even more sophisticated nonlinear machine learning methods for the analysis of GWAS, attention has recently been drawn to deep neural networks (DNN). This powerful tool for learning nonlinear relationships between an input and an output variable by transferring information through ‘a’ computing system made up of a number of simple, highly interconnected processing elements’ (34) has seen an unprecedented rise in data science (35) and created enormous progress in numerous fields, e.g. image classification (36,37), natural language processing (38), speech recognition (39) and quantum chemistry (40). DNN have been applied to the analysis of GWAS datasets (41,42), but most of the corresponding publications focus on risk prediction (28,43–45) and only very few methods have been proposed for the identification of SNP disease associations (28,46).

Romagnoni *et al.* (28) present a thorough comparison of conventional statistical approaches, traditional machine learning-based techniques and state-of-the-art deep learning-based methods in terms of both prediction rates and the identification of SNP associations on a Crohn’s disease immunochip dataset. Classification performances of numerous methods (Lasso as a reference, penalized logistic regression, gradient boosted trees, DNNs) were compared and found to be similar for most methods (linear and nonlinear) implicating potentially ‘limited epistatic effects in the genetic architecture’ (28). However, when investigating the associated genetic regions identified by the different methods, machine learning and deep learning-based methods were indeed found to provide new insights into the genetic architecture of the trait. Romagnoni *et al.* (28) achieved this by applying the concept of explainable AI, which is an emerging field of AI that has been gaining importance recently (47). It refers to techniques, which open the so-called ‘black box’ of machine learning methods and reveal the processes underlying their decisions so that the results can be better understood. The explanation method used by Romagnoni *et al.* (28)—permutation feature importance (PFI)—is a generalized, model-agnostic approach and more sophisticated methods specifically tailored to DNN are available. To the best of our knowledge, deep Taylor-based explanation techniques (48) have not yet been applied in the field of GWAS and we propose to adopt layer-wise relevance propagation (LRP) (49,50) for the analysis of such data. LRP is a direct way to compute feature importance scores and has been applied very successfully in numerous data science problems to explain decisions of DNNs (51,52). Instead of basing the importance score of a SNP on the data of that SNP alone, correlation structures and possible interactions are automatically taken into account.

To make LRP applicable as an explanation method for GWAS data, we use a very promising, well-performing machine learning-based method, called COMBI (31), as a starting point for our deep learning-based approach. COMBI is a two-step method, which first calculates a relevance score for each SNP by training a support vector machine (SVM) (53) for the classification of subjects based on their genetic profile. Using the learned SVM weights as an indicator of importance, COMBI selects the highest scoring SNPs as a subset to put into multiple hypothesis testing. This approach was shown to outperform other combinatorial approaches and a number of purely statistical analysis tools. The method we propose here can be viewed as an extension of the COMBI method (31) replacing the rather simple prediction step of an SVM with a more sophisticated deep learning method and using the concept of explainability to extract SNP relevance scores via LRP.

**Figure 1.**
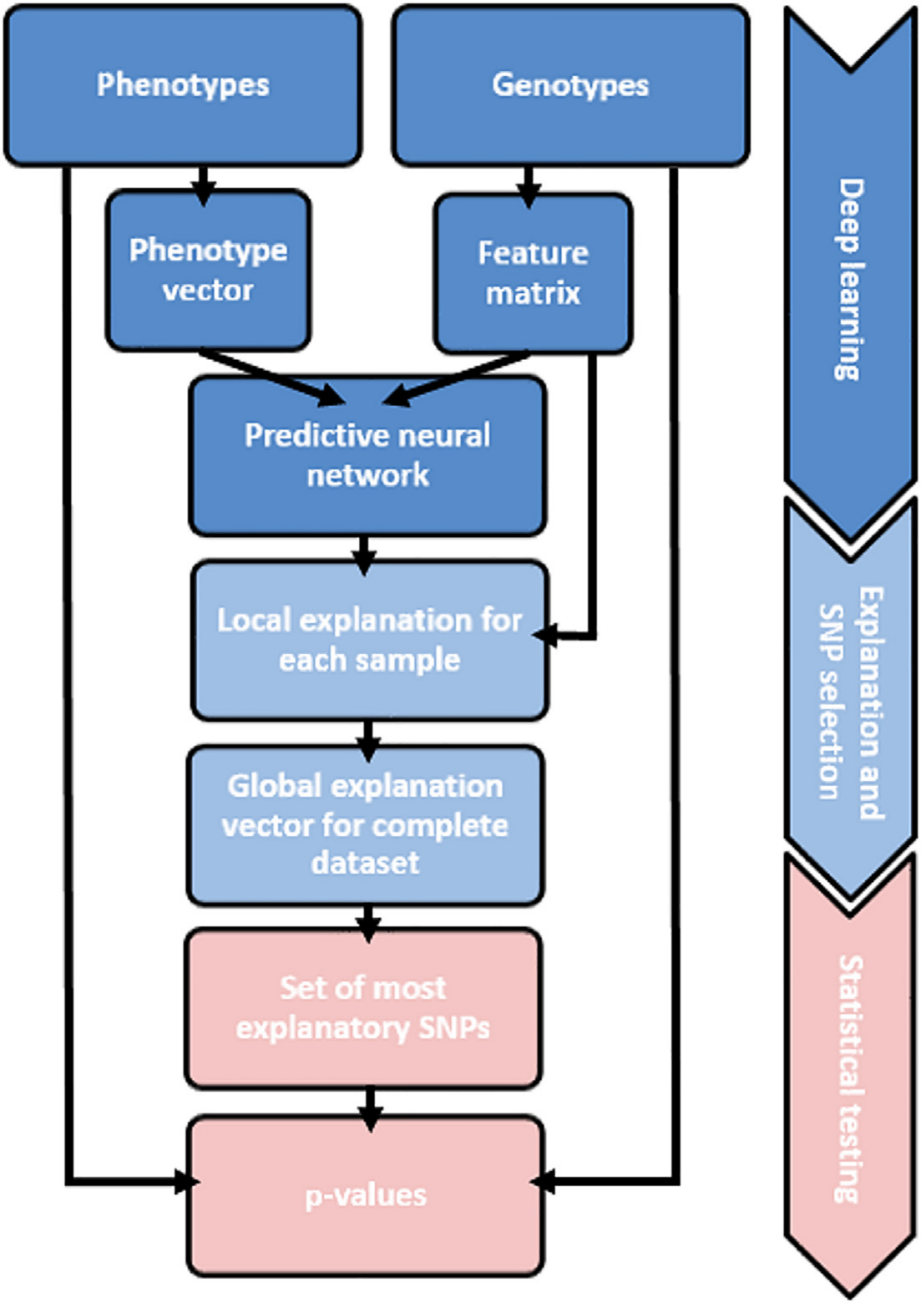
Overview of the DeepCOMBI method. Receiving genotypes and phenotypes of a GWAS as input, the DeepCOMBI method first applies a deep learning step to train a DNN for the classification of subjects. Afterward, in the explanation step, it selects the most relevant SNPs by applying LRP to calculate relevance scores for each SNP. Finally, for this set of most relevant SNPs, DeepCOMBI calculates *P*-values and corresponding significance thresholds in a statistical testing step. This figure is an adjusted version of Figure 1 presented by Mieth *et al.* (31).

We propose a deep learning-based approach for the identification of SNP phenotype associations and call the novel method DeepCOMBI (see Figure 1). The three-step algorithm consists of

1. a deep learning step where we train a DNN for classifying individuals based on their SNP data;
2. an explanation step where we calculate SNP relevance scores by applying LRP and reduce the number of SNPs by selecting only the most explanatory SNPs; and
3. a statistical testing step where only the SNPs selected in step 2 are tested for statistically significant association with the trait under investigation.

The main motivation behind DeepCOMBI is to harness the immense potential of sophisticated, state-of-the-art artificial intelligence (AI) methods to examine complex and potentially nonlinear structures in high-dimensional data by applying the concept of DNNs to GWAS in the first step of the algorithm. Subsequently, in step 2, DeepCOMBI identifies a set of SNPs that have high effects on the classification result of the DNN (either individually or in combination with other SNPs and not due to correlation structures) by calculating an explanation score for each SNP that reflects its contribution to the final classification decision. The third and last step assigns individual *P*-values to all selected SNPs and quantifies their relevance with a permutation-based significance threshold.

Figure 1 gives an overview of the overall workflow of the DeepCOMBI method, which is described in detail in the Materials and Methods section. DeepCOMBIs performance on both controlled generated datasets as well as on a 2007 GWAS dataset of seven common diseases (14) is presented in the Results section. We show that DeepCOMBI compares favorably to a number of competitor methods in terms of both classification accuracy as well as SNP association prediction when validated with all associations reported within the GWAS catalog accessed in 2020. A thorough discussion of the results and all related machine learning work is given in the Discussion section. An implementation of the DeepCOMBI method in Python is available on GitHub at https://github.com/AlexandreRozier/DeepCombi.

## MATERIALS AND METHODS

The proposed method applies deep learning and the concept of explainable AI to GWAS data and enables the identification of SNPs that are associated with a given trait with statistical significance. A graphical representation of the method is given in Figure 1. The method is based on a deep learning step that trains a DNN for the classification of GWAS subjects into their respective phenotype class. Using LRP as a post-hoc explanation method, we access the relevancies of all SNPs regarding each individual classification result. The obtained SNP relevance scores are used to select the subset of most important SNPs to test for association in the final multiple testing step.

In the following sections, we describe the statistical problem, which is investigated in a GWAS, present the proposed method in detail and specify the experimental setup of performance assessments on generated synthetic data and a real-world application of a known GWAS dataset.

### Problem setting

A GWAS investigates the observed genotypes $x = ( {{x_{ij}}} ){\;_{1 \le i \le n,1 \le j \le d}} \in {\Re ^{n \times 3d}}$ of *d* SNPs and *n* subjects labeled with the corresponding phenotypes $y = ( {{y_1}, \ldots ,{y_n}} )$. Both the genotypic information in SNP $j$ of subject $i$ and the phenotypes are encoded in a binary way, where ${x_{ij}} \in \{ {( {1,0,0} ),( {0,1,0} ),( {0,0,1} )} \}$ represents the number of minor alleles and ${y_i} \in \{ {0,1} \}$ is the binary label separating controls from cases.

The null hypothesis of a conventional single-locus test is that there is no difference between the trait means of any genotype group, which would indicate that the genotype at SNP *j* is independent of the phenotype under investigation (54). Via a chi-square test, RPVT calculates a *P*-value$\;{p_j}$ for each SNP *j* and declares it significantly associated with the phenotype if$\;{p_j} \le {t^*}$. The threshold ${t^*}$has to be chosen carefully as the significance level α in the case of a single test and adjusted if multiple tests are being conducted to bound the family-wise error rate (FWER), i.e. the probability of at least one false-positive test result, to α. Bonferroni correction is the most straightforward way to take multiplicity into account by setting ${t^*} = \frac{\alpha }{d}$ (61).

The individual RPVT *P*-value for the association of the *j*-th SNP only depends on ${x_{*j}}$ and thus disregards any possible correlations and interactions with other SNPs. Additional information can be yielded by applying machine learning-based prediction methods, which use the information of the whole genotype and calculating *P*-values only for the SNPs that were of importance in the decision process of such machines.

### DeepCOMBI

Combining the concepts of DNNs, explanation methods and statistical testing, we propose a novel algorithm consisting of the following three steps:

1. **Deep learning:** Given the genotypes $x{\rm{}} = ( {{x_{ij}}} ){\rm{\;}}$ and the corresponding phenotypes $y{\rm{}} = ( {{y_i}} ){\rm{}}$ of a GWAS, a DNN is trained for phenotype prediction.
2. **Explanation and SNP selection:** A subset of SNPs is selected by applying LRP as an explanation method for each individual prediction and averaging the absolute values of the resulting explanations to compute global prediction relevance scores ${r_1}, \ldots ,{r_d}$. The relevance scores are processed through a moving average filter with window size l and, given a predefined upper bound $k \in \{ {1, \ldots ,d} \}$ for the number of informative SNPs, we select the *k* most relevant SNPs based on *r*.
3. **Statistical testing:** A hypothesis test is performed for all SNPs selected in the previous step to compute the *P*-values of those SNPs, while the *P*-values of all other SNPs are set to one. Via a permutation-based threshold calibration and given an FWER level $\alpha$, we decide that SNP j is associated with the trait if ${p_j} < {t^{\rm{*}}}$, where ${t^{\rm{*}}} \equiv {t^{\rm{*}}}( {k,\alpha } )$ is chosen as the α-quantile of the permutation distribution of the *k* smallest *P*-values.

The proposed algorithm can be viewed as an extension of the COMBI method (31), a two-step method including an SVM step and a statistical testing step. We replace the former with state-of-the-art deep learning methods and explanation techniques. The above steps are presented in detail in the following sections.

#### The first step of DeepCOMBI—Deep learning

The first step of the proposed method consists of constructing and training a well-performing DNN for the prediction of the phenotypes $y = ( {{y_i}} )\;$of a GWAS, given the corresponding genotypes $x = ( {{x_{ij}}} )$. Selecting a DNN architecture is often critical for achieving good performance for a specific, in this case, SNP-based classification task. Montaez *et al.* (43) developed a 2-class DNN for the classification of polygenic obesity and have successfully shown its performance to be superior to numerous competitor methods. Romagnoni *et al.* (28) have compared the performance of similar architectures and have presented a detailed review of the best design choices for a DNN on a Crohn’s disease dataset. Taking inspiration from the conclusions of both of these works and having checked performances on synthetic datasets, we use an architecture of two fully connected layers with 64 neurons and ReLU activations and a dense softmax output layer with two output nodes. To improve validation accuracy by reducing overfitting, each hidden layer is followed by a dropout layer with a dropout probability of $\phi$.

The loss function to be optimized in the training process is based on the classic cross-entropy loss. Aiming for good generalization to unseen samples and in order to avoid overfitting despite the large number of model parameters, the binary cross-entropy loss is coupled with an L1-L2 mixed regularization term:

$$\begin{eqnarray*} loss &=& \mathop \sum \limits_i \left( {{y_i}*log\left( {\widehat {{y_i}}} \right) + \left( {1 - {y_i}} \right)*log\left( {1 - \widehat {{y_i}}} \right)} \right) \nonumber \\ && +\, \tau *\mathop \sum \limits_j {\left| {\left| {{w_j}} \right|} \right|_1} + \upsilon *\mathop \sum \limits_k {\left| {\left| {{w_k}} \right|} \right|_2} \end{eqnarray*}$$

with ${y_i}$ being the ground-truth label, $\widehat {{y_i}}$ the predicted class which depends on the learned parameters *w* of the DNN and $\tau ,\upsilon >0$ the regularization parameters. This loss function contributes to the ability of the network to avoid overfitting by minimizing the trade-off between small errors on the data on the one hand and small L1 and L2 norms of the vector *w* on the other hand. Adam (55) is used as an adaptive learning rate optimizer to minimize the given loss function.

To overcome limitations due to imbalanced datasets, class weights were calculated according to the class frequencies and used to direct the DNN to balance the impact of controls and cases.

Once the parameters *w* of the DNN have been trained by optimizing the above learning problem, the network is able to predict the phenotype of any unseen genotype *x*. Regarding this binary classification problem, the output node with the highest score represents the predicted phenotype.

In a preprocessing step, the data are centered and scaled by subtracting the global mean and dividing by the global standard deviation. To minimize computational effort and limit the number of model parameters in the DNN, a *P*-value threshold $\kappa$ can be applied in order to only select SNPs with *P*-values smaller than $\kappa$ to be used for training. This preprocessing step can be applied to large datasets when limited computational resources are available. While all datapoints remain in the dataset, the number of features of the dataset is decreased to only train the DNN on the SNPs with a *P*-value smaller than the threshold κ. The set of features remains this size for all further steps (i.e., DNN training, LRP explanation, SNP selection, etc.) and all SNPs with a *P*-value above the threshold automatically get assigned a relevance score of 0 and are no longer candidates for the DeepCOMBI method. For a discussion of the potential effects of this feature selection step and potential adoptions to improve it, please refer to the Discussion section below.

#### The second step of DeepCOMBI—Explanation and SNP selection

To harness the potential of DNNs in the identification of SNP disease associations in GWAS, we now apply the concept of explainable AI. Once the DNN is fully trained, the aim is to define an importance measure that determines which loci play an important role in the determination of a phenotype. Generating relevance scores from trained DNNs can be achieved by using LRP (48–50), which consists of the following two steps: After a DNN *f* is trained on a prediction task, the prediction scores of a datapoint ${x_i}$ are computed by $f( {{x_i}} ) = {y_i}$, a forward pass through the network. Afterward, following a specific propagation rule, a single output score, i.e. the highest output score, ${y_i}$ is backpropagated successively layer-by-layer through the network until reaching the input layer. In this work, we use the $\alpha \beta$- LRP rule, where the relevance $R_s^{( {q,i} )}$ of neuron *s* in layer *q* depends on the relevance of all of its successors *t* in layer *q+1* in the following way:

$$\begin{equation*}{\rm{}}R_s^{\left( {q,i} \right)} = \mathop \sum \limits_t \left( {\alpha \frac{{{{\left( {{a_s}{w_{st}}} \right)}^ + }}}{{\mathop \sum \nolimits_s {{\left( {{a_s}{w_{st}}} \right)}^ + }}} - \beta \frac{{{{\left( {{a_s}{w_{st}}} \right)}^ - }}}{{\mathop \sum \nolimits_s {{\left( {{a_s}{w_{st}}} \right)}^ - }}}} \right) \times R_t^{\left( {q + 1,i} \right)}\end{equation*}$$

where ${a_s}$ denotes the activation of neuron *s*, and ${w_{st}}$ is the weight between the two neurons *s* and *t*. This rule allows us to weigh the positive and negative contributions of neurons *t* to their predecessor *s* differently by $\alpha$ and $\beta$.

Once the input layer ${R^{( {0,i} )}} \in {\Re ^{3*d}}$ is reached, a relevance score ${\rho _{ij}}$ of SNP *j* in subject *i* is attributed to each dimension of ${x_i}$ with

$$\begin{equation*}{\rho _{ij}} = {{\left( {\mathop \sum \limits_u R_u^{\left( {0,i} \right)}} \right)} \mathord{\left/ {\vphantom {{\left( {\mathop \sum \limits_u R_u^{\left( {0,i} \right)}} \right)} 3}} \right. } 3}.\end{equation*}$$

Since the original relevance vector ${R^{( {0,i} )}}$ contains three values for each one-hot encoded location, it is converted back to size *d* by averaging over the three nodes $u \in \{ {( {j \times 3} ) - 2,( {j \times 3} ) - 1,( {j*3} )} \}$ corresponding to SNP j in the input layer.

Note that all relevance scores ${\rho _{ji}}$ will be positive since a softmax output layer with two output nodes for the binary classification problem was used and only the highest of the two output activations was backpropagated.

${\rho _{ij}}$ now demonstrates to which extent the dimension *j* of ${x_i}$ plays a role in the classification decision $f( {{x_i}} )$ and can be used to uncover the most relevant SNPs for prediction. Note, however, that LRP is applied individually to each datapoint *i*. By averaging the values of all individual LRP explanations ${\rho _{ij}}\,\,{\rm{of}}\,\,{\rm{SNP}}\,\,j,$ we propose to generate a global explanation

$$\begin{equation*}{\rm{}}{r_j} = \left( {\mathop \sum \limits_{i = 1}^n {\rho _{ij}}} \right)\mathord{\left/ {\vphantom {{\left( {\mathop \sum \limits_u R_u^{\left( {0,i} \right)}} \right)} n}} \right. } n\end{equation*}$$

which is independent of datapoints. The relevance scores of one sample sum up to the activation value of the output prediction, which means that datapoints classified with low certainty will also have a small impact on the global explanation. Intuitively, the global LRP score *r_j_* of each SNP *j* can now be interpreted as a measure of relevance regarding the prediction. The higher *r_j_*, the greater the influence of locus *j* on the decision process of the DNN.

To achieve better performance, Mieth *et al.* (31) suggested that SNP relevance scores should be filtered before using them to select the highest scoring locations. Hence, the LRP relevance score vector *r* is post-processed through a *p-*th-order moving average filter, that is:

$$\begin{equation*}r_j^{new}: = \sqrt[p]{{\mathop \sum \limits_{h = max\left( {1,j - \left( {l - 1} \right)/2} \right)}^{min\left( {d,j + \left( {l - 1} \right)/2} \right)} {{({r_h})}^p}}}\end{equation*}$$

where $l \in 1, \ldots ,d$ denotes the window size *l* and $p \in ] {0,\infty } [$. We have now generated relevance scores showing which SNPs played an important role in the classification decision and can use them for the selection of promising locations. For the next step of DeepCOMBI, we choose to test all SNPs with the *k* largest values of the scores $r_j^{new}$ and eliminate all SNPs with lower relevance.

#### The third step of DeepCOMBI—Statistical testing

The Statistical testing step of the DeepCOMBI method is directly derived from the second step of the COMBI method (31). A ${\chi ^2}$hypothesis test is performed for each of the *k* SNPs selected in the LRP explanation step and the *P*-values for all other SNPs are set to one. To identify statistically significant associations, a *P*-value threshold ${t^*}$ is calibrated to control the *FWER* for multiplicity by applying the permutation procedure proposed by Mieth *et al.* (31). They developed an extension of the Westfall and Young procedure (56). A thorough discussion and derivation of the method, its assumptions and validity generally and in this specific application can be found here (56–58) and here (31), respectively. We estimate the distribution of *P*-values under the global null hypothesis of no informative SNPs by repeatedly assigning a random permutation of the phenotypes to the observed genotypes and applying the complete workflow of the DeepCOMBI method to save the resulting *P*-values of the *B* Monte Carlo repetitions (31). The empirical lower $\alpha$-quantile of the smallest of these *P*-values is then a valid choice for ${t^*}$ in the sense that the *FWER* for the entire procedure is bounded by $\alpha$(31). In contrast to the Bonferroni threshold calibration, this procedure takes all dependencies in GWAS datasets caused by strong linkage disequilibrium (LD) into account.

### Baselines

In order to evaluate the performance of the proposed DeepCOMBI method in comparison to competitor approaches, we select a set of representative baseline methods. RPVT is chosen as the most widely used traditional, purely statistical testing approach. As a machine learning-based method and the methodological background of DeepCOMBI, we select COMBI as the main competitor method we aim to succeed in terms of performance. Since the COMBI method was shown to outperform other combinatorial machine learning-based approaches (6,58,59) and a number of purely statistical analysis tools (21,27) on the same datasets evaluation methods used here, there is no need to compare to all of those methods again.

#### RPVT as a baseline

Raw *P*-value thresholding (RPVT) is a statistical framework traditionally used in GWAS for identifying significant associations between SNPs and traits. The single-locus null hypothesis to be tested states that the SNP at locus *j* is independent of the binary trait of interest, i.e. that there is no correlation between this particular SNP and the development of the disease under investigation. A standard statistical test for this hypothesis is the ${\chi ^2}$-test (60), which tests for independence of the two multi-level variables genotype (three different levels: 0, 1 or 2 minor alleles) and phenotype (two different levels: case or control) by calculating the test statistic

$$\begin{equation*} {\hat \chi ^2} = \mathop \sum \limits_{\zeta ,\pi } \frac{{( {O_{\zeta ,\pi}} - {E_{\zeta ,\pi }} )}^2}{{E_{\zeta ,\pi }}} \end{equation*}$$

where ${O_{\zeta ,\pi }}$ and ${E_{\zeta ,\pi }}$ are the observed and expected frequencies of genotype $\zeta$ in combination with phenotype $\pi$. To compute a *P*-value, ${\hat \chi ^2}$ is then compared to a ${\chi ^2}$ distribution with two degrees of freedom. The *P*-value then represents the probability of observing a sample statistic as extreme as ${\hat \chi ^2}$under the assumption of no association between genotype and phenotype. If it is smaller than a predefined threshold ${t^*}$, the null hypothesis is rejected and we declare the SNP under investigation to be significantly associated with the phenotype. If there was a single test to perform, ${t^*}$ would usually be equal to the significance level $\alpha = 0.05$. When performing multiple testing, however, the threshold is modified to take the multiplicity of the problem into account. The simplest method is the so-called Bonferroni correction (61), where ${t^*}$ is divided by the number of tests performed, i.e*. d*, the number of SNPs in our case, which guarantees that the FWER, the probability of one or more erroneously reported associations, is bounded by α. The Bonferroni correction works well under the assumption that all null hypotheses are independent of each other, which is not the case here. Indeed, since SNPs show high degrees of correlation through LD, the Bonferroni correction can become extremely conservative, leading to a high rate of false rejections, which is why the scientific community mostly applies a fixed threshold that remains constant for multiple GWAS. Here, based on the original publication of the data we are analyzing (WTCCC data, see the Materials and Methods section on validation datasets (14)) and the findings of Mieth *et al.* (31), we present not only the strong associations at a significance level of ${t^*} = 5x{10^{ - 7}}$ but also weak associations at ${t^*} = 1 \times {10^{ - 5}}$.

#### COMBI as a baseline

The COMBI method (31) combines machine learning with multiple hypothesis testing to improve the statistical power of GWAS. It is a two-step method including the training of an SVM (53) and using the resulting SVM weights as importance scores to select a subset of candidate SNPs for statistical testing. In the first step of COMBI, an SVM for the prediction of the unknown phenotype $y$ based on the observation of genotype $x$ is trained to determine the weight vector *w*. The following optimization problem is solved:

$$\begin{equation*}w = argmi{n_w}\left( {\left\| w \right\|_2^2 + C\mathop \sum \limits_{i = 1}^n max\left( {0,1 - {y_i}{w^T}{x_{i*}}} \right))} \right)\end{equation*}$$

where *C* is the regularization parameter that controls the trade-off between a small norm of *w* and a small prediction error of the machine. After training, the weight vector *w* is filtered and interpreted as an importance score to determine which loci play an important role in the decision process of the SVM. A ${\chi ^2}$ test is performed only on the SNPs with the highest scores while all other *P*-values are set to one. The same permutation test procedure as described in the Materials and Methods section about the multiple testing procedure of DeepCOMBI is applied to define a significance threshold ${t^*}$.

#### Raw SVM weights and LRP scores without statistical testing as baselines

Instead of interpreting the SVM weights from COMBI and the LRP scores from DeepCOMBI as relevance scores to select a subset of SNPs to calculate *P*-values for, this step can be skipped to use the raw SVM and LRP scores as a test statistic. For evaluation, the vector of raw SVM weights and LRP scores can be treated like the vector of *P*-values of RPVT, COMBI and DeepCOMBI to calculate performance curves. We compare DeepCOMBI to these baseline methods of raw relevance scores and RPVT to show that only the combination of machine learning / deep learning and multiple testing shows the desired performance increase, which cannot be achieved individually by one of the components.

In an additional benchmark analysis, we compare the SNP discoveries of DeepCOMBI to those of Lippert *et al.* (21,27) who applied linear mixed models (LMMs) to the seven 2007 WTCCC datasets (which are described in more detail below) and explicitly take confounding factors such as population structure, family structure and relatedness into account.

### Validation datasets

#### Validation on generated datasets

To create a realistic but controlled environment where the ground truth labels of a dataset, i.e. the SNPs that are indeed linked to the disease, are known, we generate semi-synthetic data for a first evaluation of DeepCOMBI and the baseline methods from above. We follow the instructions for the creation of such GWAS datasets proposed by Mieth *et al.* (31). The basic concept is to take an ensemble of real genotypes and generate a synthetic phenotype for each subject according to a specific rule. With this method, the underlying architecture of the genome, including, for example, genetic LD and correlation structures, is kept intact while control over the phenotypic labels is gained at the same time.

We use the WTCCC dataset (14) described in more detail below and randomly select 300 subjects of the Crohn’s disease dataset. We draw a random block of 20 consecutive SNPs from chromosome 1 and a random block of 10 000 consecutive SNPs from chromosome 2. The former block represents the informative SNPs and is placed in the middle of the 10 000 uninformative SNPs. Synthetic phenotypes are now generated only based on one of the informative SNPs (at position 5010) according to the following phenotype probability distribution:

$$\begin{equation*}P\left( {{Y_i} = + 1|\;{X_{i,*}} = {x_{i,*}}} \right) = \frac{1}{{1 + {e^{ - \gamma \left( {{x_{i,5010}} - median\left( {{x_{*,5010}}} \right)} \right)}}}}\end{equation*}$$

where $\gamma$ is an effect size parameter, ${x_{i,*}}$ is the allele sequence in nominal feature encoding (i.e. ${x_{ij}}$ is the number of minor alleles in SNP $j$ of subject $i$) and ${Y_i}$ is the generated phenotype of subject *i*. Basing the label of a subject on the SNP at position 5010 alone will create associations to all 20 informative SNPs and typical tower-shaped *P*-value formations in the resulting Manhattan plots because there are real covariance structures and LD within the 20 informative SNPs. At the same time, the tower structure is limited to those 20 informative positions because there are no correlations of those 20 SNPs with the surrounding 10 000 noise SNPs coming from chromosome 2. The random generation process will also ensure that the datasets will have associations of different strengths to the 20 informative SNPs. The complete data generation process is repeated to generate 1000 datasets. DeepCOMBI and all baseline methods are applied to each dataset with an 80:20 class balanced split in training and test data. The prediction results on the test data are evaluated with the known ground truth of only 20 informative SNPs at the positions 5000–5020 and the corresponding performance can be measured in terms of the number of true and false positives for each method.

**Figure 2.**
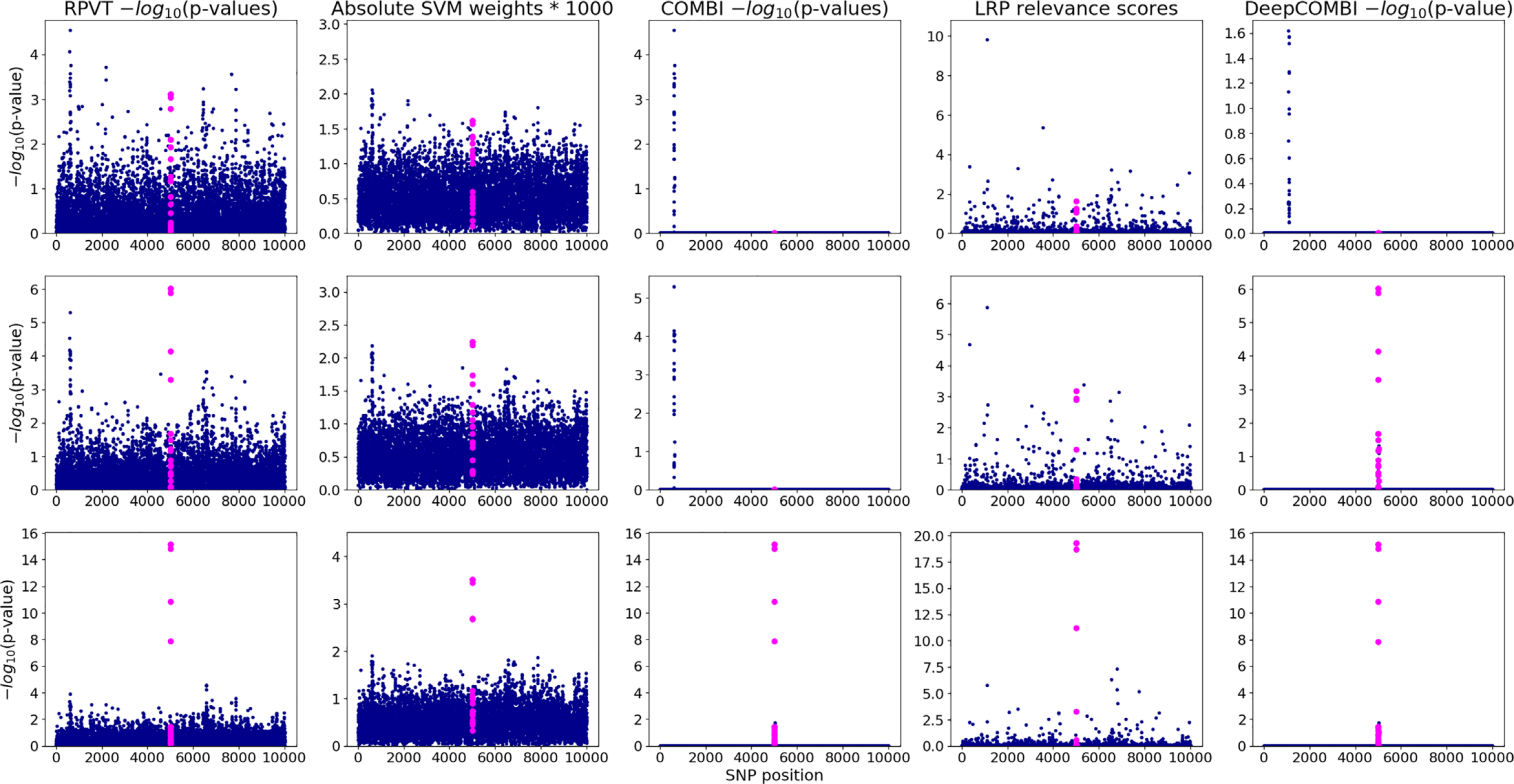
Three exemplary generated datasets and the corresponding COMBI and DeepCOMBI results. We present the results of three exemplary replications: one with weak (first row), one with medium (second row) and one with strong (third row) association of the 20 informative SNPs at position 5001–5020 (highlighted in all subfigures). Standard RPVT *P-*values are plotted in the first column of subfigures. Absolute SVM weights and corresponding *P-*values of the COMBI method are shown in the second and third columns. Finally, LRP relevance scores and the corresponding *P-*values of DeepCOMBI are presented in the fourth and last column.

Since we have adopted the data generation procedure from Mieth *et al.* (31), let us point out that we do not perform a full investigation on its assumptions and its ability to produce realistic GWAS datasets here. Please refer to Mieth *et al.* (31) and in particular its Supplementary File where Chapter 3 is dedicated to all necessary experiments and corresponding results can be found. To assess the performance of the data generation procedure here, we include Manhattan plots of three exemplary datasets in Figure 2. To sum up, the experiments performed in Mieth *et al.* (31), they investigated the effect size parameter $\gamma$ and identified $\gamma = 6$ to be the value that yields the most realistic tower structures. This investigation was based purely on the RPVT *P*-values of the resulting datasets and the level of difficulty in the generated datasets was evaluated based on these *P*-values alone.

In addition, it was shown that an effect size of 6 was the value where the permutation test procedure produced the anticipated family-wise error rate of 0.05. Mieth *et al.* (31) provide analyses assessing the difficulty of the problem under investigation by estimating it with the true-positive rate of RPVT. Extraordinary cases where the generated datasets are either exceptionally hard or exceptionally easy are also investigated. Furthermore, the effect of misspecifications with effect sizes too high or too low is examined.

#### Validation on WTCCC data

For evaluation on real-world genomic data, the performance of DeepCOMBI was assessed on the Wellcome Trust Case Control Consortium phase 1 dataset, released in 2007^14^, featuring the genotypic information on 500 000 SNPs of 17 000 British subjects. With 3000 shared controls and 2000 case samples for seven major human diseases (Crohn’s disease [CD], type 1 diabetes [T1D], type 2 diabetes [T2D], coronary artery disease [CAD], hypertension [HT], bipolar disorder [BD] and rheumatoid arthritis [RA]), it was a landmark study both in terms of sample size and dimensionality at the time of its publication. For our analysis, a case–control dataset for each disease was created, removing all SNPs and samples that did not fulfill the quality control criteria provided in the original WTCCC paper. In agreement with the lack of inter-chromosomal LD and the findings of Mieth *et al.* (31), who showed no significant performance increase with genome-wide training, the DeepCOMBI method and all baseline methods were applied to each chromosome separately.

For evaluation purposes, the concept of replicability was applied proposed by Mieth *et al.* (31). Since the true underlying genetic architecture of the given traits, i.e. the sets of informative SNPs for each disease, is unknown, an approximation of the truth was created by employing the GWAS catalog (62) and examining the results of the 13 years of independent studies after the WTCCC dataset was published. To evaluate the reported finding of a method (DeepCOMBI or competitor), the GWAS catalog (accessed on 30 July 2020) is inquired for that SNP and all SNPs in LD ([*R*^2^ > 0.2] according to PLINK LD calculations (63)) within a 200 kb window around that SNP. If an association with the disease with *P*-value <10^–5^ of the SNP itself or the SNPs in LD was reported by at least one independent GWAS published after the WTCCC study, the reported SNP is counted as a true positive finding. In contrast, all SNPs that were not replicated count as false negatives.

### Parameter selection

The application of DeepCOMBI requires the determination of a number of free parameters. In the following sections, we present the selected optimal parameter values and describe the process of finding them for the different datasets under investigation.

#### Parameter selection on generated datasets

For the generation process of semi-synthetic datasets, all parameters were selected according to the information given by Mieth *et al.* (31). Most importantly, the effect size parameter was set to $\gamma = 6$.

When applying the DeepCOMBI method to the generated datasets, we studied the effect of all hyperparameters on the performance of the DNN. An accuracy-based random grid search with a stratified split in 90% training and 10% testing data was conducted. Here, we present the selected most successful values and the investigated parameter intervals in parentheses:

- number of neurons per dense hidden layer nn = 64 [2, 4, 8, 16, 64],
- L1 regularization coefficient $\tau = 0.0001$ [0, 1e-6, 1e-5, 1e-4, 1e-3,1e-2, 1e-1],
- L2 regularization coefficient $\upsilon = 0.000001$ [0, 1e-6, 1e-5, 1e-4, 1e-3,1e-2, 1e-1],
- dropout rate $\phi = 0.3$ [0.3, 0.5],
- learning rate $\eta = 0.01$ with learning rate reduction on a plateau with factor 0.7125 after 50 epochs of no improvement and
- number of epochs e = 500 [100, 500, 1000].

A few different parameter values of the $\alpha \beta$- backpropagation rule were manually investigated on exemplary datasets. By visually inspecting the resulting LRP vectors and their corresponding DeepCOMBI *P*-values, the combination of $\alpha = 1$ [0, 1, 2] and $\beta = 0$ [0, 1, 2] was found to be best.

For post-processing the global relevance scores and selecting the most relevant SNPs, we assumed that the most successful values found by Mieth *et al.* (31) would also be a good choice for our method. Hence, we set the window size of the moving average filter to *l* = 35, the norm parameter of the moving average filter to *P* = 2 and the SNP selection parameter to *k* = 30. These values were found to be in agreement with the biological background of the data, e.g*. l* = 35 reflects the reach of LD along a genetic sequence (31).

#### Parameter selection on WTCCC data

To choose hyperparameters for the DNN trained on WTCCC data in the first step of DeepCOMBI, a parameter search was run on a single dataset. The Crohn’s disease chromosome 3 dataset was selected as a good representative, and an accuracy-based parameter search with a stratified split in 90% training and 10% testing data was conducted. We studied the effect of the hyperparameters on the performance of the DNN and the best performing hyperparameters were as follows (tested intervals in parentheses):

- number of neurons per dense hidden layer *nn* = 64 [2, 4, 8, 16, 64],
- L1 regularization coefficient $\tau = 0.001$ [0, 1e-6, 1e-5, 1e-4, 1e-3,1e-2, 1e-1],
- L2 regularization coefficient $\upsilon = 0.0001$ [0, 1e-6, 1e-5, 1e-4, 1e-3,1e-2, 1e-1],
- dropout rate $\phi = 0.3$ [0.3, 0.5],
- *P*-value threshold $\kappa$ = 1e-2 [1e-4, 1e-2, 1],
- learning rate $\eta = 0.00001$ [1e-7, 1e-6, 1e-5, 1e-4, 1e-3,1e-2, 1e-1],
- number of epochs *e* = 500 [100, 500, 1000].

Detailed results on the classification performance of the final training parameter settings and the corresponding evolution of training and validation measures on an exemplary dataset can be found in the Results section.

As before, we visually investigated a few different parameter values of the $\alpha \beta$ - backpropagation rule and their influence on both the resulting relevance scores and *P*-values. On the Crohn’s disease chromosome 3 dataset, the combination of $\alpha = 2$ [0, 1, 2] and $\beta = 1$ [0, 1, 2] was found to be optimal.

After manually investigating the global LRP scores and the corresponding DeepCOMBI *P-*values of the exemplary dataset (Crohn’s disease chromosome 3), we found that slightly different settings than for the analysis of the generated datasets should be applied for post-processing the relevance vectors and selecting the most relevant SNPs. Namely, the window size of the moving average filter should be set to *l* = 21 and the SNP selection parameter should be increased to *k* = 200. The need for a decreased filter size and an increased number of selected SNPs might be caused by the application of the *P*-value-based preselection step for limiting the number of model parameters, which is only applied to the real dataset and not the generated datasets.

To determine the value of the significance level $\alpha$ to be used in the permutation test procedure of the last steps DeepCOMBI, we follow the recommendations of Mieth *et al.* (31), who calculated the empirical distribution of *P*-values using the Westfall-Young (56) procedure and determined the error level that the RPVT threshold of ${t^*} = 1 \times {10^{ - 5}}$ corresponds to. For a valid comparison to both the original WTCCC study as well as the COMBI publication, we employ the same significance levels.

All free parameters of the COMBI method, e.g. the SVM optimization parameter *C*, were set according to the original COMBI publication (31).

### Performance metrics

To assess the performance of DeepCOMBI and the baseline methods, a number of statistical metrics were evaluated. The performances of both the intermediate step of classification (of SVMs and DNNs) and the final result of predicted informative SNPs need to be explored.

Assuming we know the ground truth, the metrics are defined as follows:

- TP = True positive; FP = False positive; TN = True negative; FN = False negative
- Accuracy = (TP + TN)/(TP + TN + FP + FN)
- Balanced accuracy = (TPR + TNR)/2
- Precision = TP/(TP + FP)
- True positive rate TPR = TP / (TP + FN)
- False positive rate FPR = FP / (TP + FN)
- Family-wise error rate FWER = P(FP > = 1)

The following performance curves and the area under these curves (AUC) will be investigated:

- Receiver operating characteristic curve (ROC): TPR versus FPR or TP versus FP or TPR versus FWER
- Precision-recall curve (PR): Precision versus TPR or Precision versus TP

### Implementation details

The DeepCOMBI method was implemented in Python and the source code is available at https://github.com/AlexandreRozier/DeepCombi. The implementation uses the DNN development library Keras (64) in combination with the LRP library iNNvestigate (65).

## RESULTS

In the following section, we present the results of the proposed DeepCOMBI method evaluated on generated as well as on real-world data. Performance in terms of both classification accuracy and SNP prediction is examined in comparison to a number of baseline methods, which are presented in full detail in the Materials and Methods Section. As evaluation criteria, we report prediction accuracy for the classification step and *FWER*, precision and *TPR* for the SNP selection step. See the Materials and Methods section above for a detailed description of the evaluated performance metrics.

### Results on generated datasets

Here, we report our results averaged over the 1000 data sets generated in the simulation process described in the Materials and Methods section (‘Validation on generated datasets’). We show that, on these data sets, DeepCOMBI performs better than the traditionally used method for analyzing GWAS, RPVT, and its main competitor, the COMBI method.

#### Prediction performance on generated datasets

The first steps of both DeepCOMBI and COMBI consist of training a learning algorithm for the classification of all subjects into their respective phenotypic group given their genotypic information. Since all following steps depend on the performance of these classifiers, high prediction accuracy is crucial. On the generated datasets, the SVM (as part of the COMBI method) achieves 59% accuracy on average and 54% balanced accuracy. In comparison, the DNN (as part of the DeepCOMBI method) performs significantly better with an average of 74% classification accuracy and also avoids negative effects of unbalanced datasets more effectively by applying class weights in the DNN training (74% balanced accuracy). Accuracy scores and additional information are given in Table 1. Following these promising intermediate results, in the next section, we investigate whether the entire workflow of the DeepCOMBI method can also outperform the baseline methods.

**Table 1.** Classification performance on generated datasets. Summary statistics of the classification accuracy of the SVM (as in the first step of COMBI) and the DNN (as in the first step of DeepCOMBI) are presented. Values corresponding to accuracy and balanced accuracy in parentheses are given

	Mean accuracy (balanced accuracy)	Standard deviation of accuracy (balanced accuracy)	Minimum of accuracy (balanced accuracy)	Maximum of accuracy (balanced accuracy)
**SVM (as in COMBI)**	0.59 (0.54)	0.05 (0.06)	0.41 (0.35)	0.76 (0.71)
**DNN (as in DeepCOMBI)**	0.74 (0.74)	0.07 (0.07)	0.55 (0.50)	0.97 (0.98)

#### SNP selection performance on generated datasets

To compare the relevance scores and *P*-values obtained with the novel LRP-based method to those derived from the SVM weights in the COMBI method, we look at three exemplary synthetic datasets and the corresponding results (see Figure 2). They can be distinguished by the level of association of the 20 informative SNPs with the phenotype. In the first column of subfigures, the strength of association for each replication at positions 5001–5020 is shown in the corresponding RPVT Manhattan plots. While the first row of subfigures represents a replication with very weak associations (small tower), the second has a moderate association (medium-sized tower) and the third shows a very strong association (large tower). In the second and third columns, the raw SVM weights and LRP scores are shown. It can be seen that LRP yields clearer relevance distributions in comparison to the SVM-based method. Even with the huge number of parameters, the DeepCOMBI explanation method yields a lot less noise than the SVM weights of COMBI. This results in the COMBI method only being able to classify the very strong association correctly (third column of subfigures), while it misses the weak and moderate ones. In contrast, DeepCOMBI is successful for both the second and third replication with moderate and strong associations and only misses the very weak association (last column of subfigures). Please note that DeepCOMBI not only precisely identifies the correct informative tower but also filters out a relatively high noise tower at around position 600, which—just by chance— achieved a *P*-value $ < {10^{ - 5}}$. The method thus not only increases the probability of finding the correct tower but also, and potentially more importantly, decreases the probability of falsely selecting a noise tower (31).

**Figure 3.**
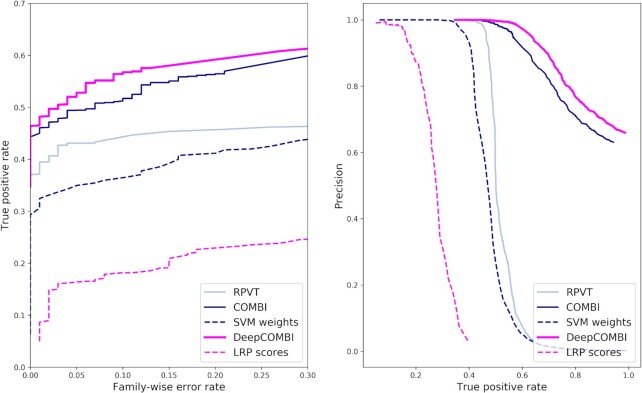
ROC and PR curves of DeepCOMBI and all competitor methods on generated datasets. Performance curves of all methods averaged over the 1000 generated datasets are shown. ROC curves are presented on the left and PR curves on the right side.

To investigate whether these exemplary findings represent a general trend, we now examine the results of all competitor methods averaged over all 1000 generated datasets. In Figure 3, the corresponding ROC and PR curves are shown. In both subfigures and for all levels of error and detection rates, DeepCOMBI consistently outperforms RPVT and COMBI in terms of power and precision. The combinatorial approaches, DeepCOMBI and COMBI, also perform better than their individual components of a machine learning algorithm (SVM or DNN with LRP) and a multiple testing step (RPVT). This can be deduced from the fact that RPVT, as well as the other two baseline methods of directly thresholding the raw LRP scores and SVM weights separately, cannot achieve the same performance as their combinations (i.e. DeepCOMBI and COMBI).

### Results on WTCCC data

#### Prediction performance on WTCCC data

**Figure 4.**
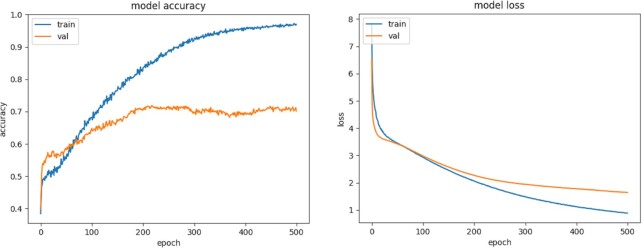
Training and validation metrics on an exemplary WTCCC dataset. Evolution of model metrics during DNN training on Crohn’s disease chromosome 3 dataset in 500 epochs. Model accuracy on both training and validation datasets is shown on the left and model loss (also on training and validation data) on the right.

In the first step of DeepCOMBI, a DNN is trained and we present the evolution of both training and validation loss and accuracy during training on an exemplary dataset (i.e. the Crohn’s disease chromosome 3 dataset) in Figure 4. Overfitting is avoided and both training and validation accuracy increase during training and reach an optimum at the end of the training process (to be seen on the left). Good generalization can also be seen on the right where the model loss decreases with each epoch on both training and validation data.

**Figure 5.**
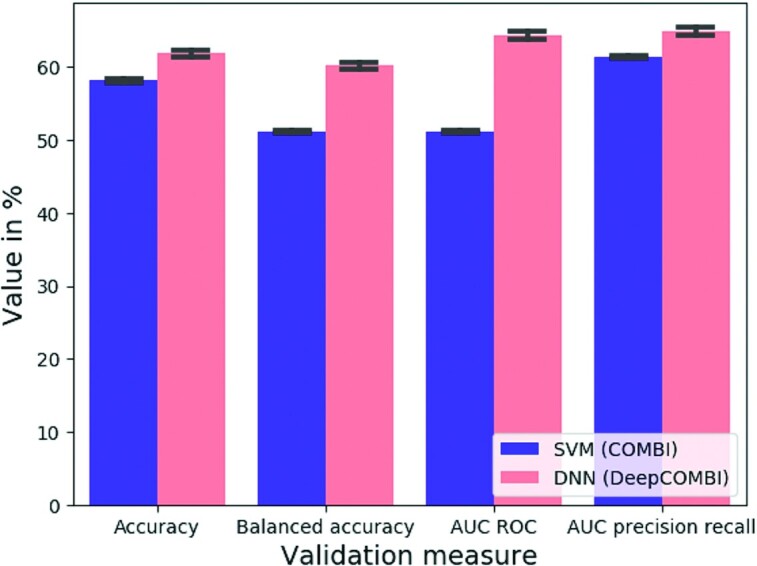
Classification performance on WTCCC data. Mean validation measures of SVM (as in the first step of COMBI) and DNN (as in the first step of DeepCOMBI) averaged over all diseases and chromosomes are given with standard deviation. All datasets were split into 80% training and 20% validation data.

We now investigate the performance of the DNN on all diseases and chromosomes. Figure 5 shows that the DNN of DeepCOMBI performs consistently better than the SVM of COMBI in terms of all four validation metrics described in the Materials and Methods section under ‘Performance metrics’.

#### SNP selection performance on WTCCC data

**Figure 6.**
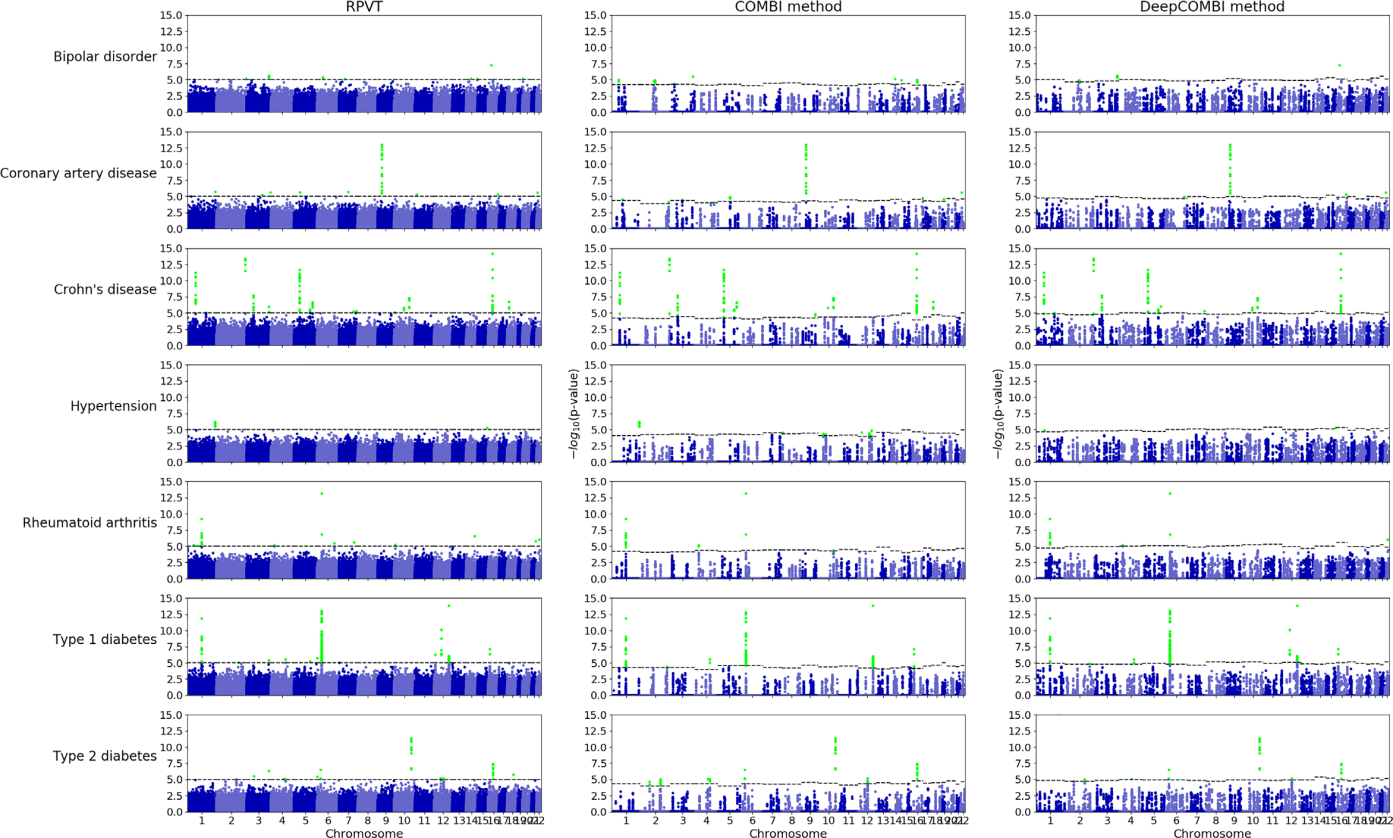
Manhattan plots for WTCCC data. The negative logarithmic ${\chi ^2}$test *P*-values are plotted against position on each chromosome for all seven diseases. Results from the standard RPVT approach, the COMBI method and the DeepCOMBI method are shown. Thresholds indicating statistical significance are represented by dashed horizontal lines and significant *P*-values are highlighted. Please note that the *y*-axes of all plots have the same limits (0–15) to enable direct comparison.

In Figure 6, we present the results of the traditional RPVT approach, the COMBI method and the DeepCOMBI applied to the seven diseases of the WTCCC 2007 dataset. In each corresponding Manhattan plot, the negative logarithmic *P*-values of all SNPs at a given position in a chromosome are shown. While RPVT assigns *P*-values smaller than one (i.e. nonzero in the plots on a logarithmic scale) to all SNPs and, in consequence, produces a lot of statistical noise, both COMBI and DeepCOMBI discard most SNPs (i.e. assign *P*-value one, i.e. zero in the plot on a logarithmic scale) and hence reduce the level of noise significantly. The COMBI method selects 100 SNPs with high SVM weights per chromosome and DeepCOMBI chooses 200 SNPs with high LRP scores. In all plots, the significance threshold ${t^*}$ is represented by dashed horizontal lines and all statistically significant SNP associations are highlighted. Please note that in the case of RPVT, the threshold is constant at ${t^*} = 1 \times {10^{ - 5}}$ (i.e. 5 in the plot) for all chromosomes. A chromosome-wise threshold was generated for both COMBI and DeepCOMBI via the permutation-based procedure described in the Materials and Methods section to match the expected number of false rejections of RPVT.

All SNPs reaching statistical significance in the permutation-based thresholding procedure of the DeepCOMBI method are presented in Table 2. Besides showing basic information (associated disease, chromosome, identifier and χ^2^*P*-value) for all of these SNPs, the fifth and sixth columns indicate whether they were found to be significant by RPVT with the application of ${t^*} = 10_{}^{ - 5}$ or by the COMBI method. To validate all findings, the seventh and eighth columns report whether—and if so in which external study—they have been found significantly associated with the given disease according to the GWAS catalog. By investigating whether the identified SNPs were discovered as significant in an independent GWAS published after the original WTCCC study, it can be determined to which extent those novel findings can be confirmed to be true associations.

**Table 2.** Significant SNPs of the DeepCOMBI method and related association details. For each SNP identifier on a specific chromosome that was found to be significantly associated with a disease by the DeepCOMBI method, we show their χ^2^ test *P*-value and indicate whether the RPVT *P*-value is < 10^–5^ (i.e. the SNP is a significant finding of RPVT), whether its COMBI *P*-value is smaller than the corresponding COMBI threshold (i.e. the SNP is a significant finding of the COMBI method) and whether the SNP has been found significant with a *P*-value < 10^–5^ in an external study with a corresponding PMID. Please note that the RPVT result in the fifth column corresponds to the χ^2^*P*-values we have calculated here, not necessarily to the original WTCCC publication, where they also investigated trend test *P*-values and potentially applied slightly different preprocessing steps. Similarly, the COMBI result in the sixth column corresponds to the re-calculations of COMBI we performed here, not necessarily to those of the original COMBI publication where slightly different results were produced due to the random nature of the permutation procedure

Disease	Chromosome	Identifier	χ^2^*P*-value	Significant in RPVT	Significant in COMBI	*P*-value < 10^–5^ in at least one external GWAS or meta-analysis	References (PMID)
**Bipolar disorder (BD)**	2	rs7570682	1.77e-05		YES	YES	21254220
	2	rs1375144	1.26e-05		YES	YES	21254220
	3	rs514636	2.53e-06	YES	YES	YES	21254220
	16	rs420259	5.87e-08	YES	YES	YES	21254220
**Coronary artery disease (CAD)**	6	rs6907487	2.92e-05			YES	17634449
	9	rs1333049	1.12e-13	YES	YES	YES	17634449
	16	rs8055236	5.32e-06	YES	YES		
	22	rs688034	2.75e-06	YES	YES		
**Crohn’s disease (CD)**	1	rs11805303	6.35e-12	YES	YES	YES	17435756
	1	rs12037606	1.02e-05			YES	17554261
	2	rs10210302	4.52e-14	YES	YES	YES	23128233
	3	rs11718165	2.04e-08	YES	YES	YES	21102463
	5	rs6596075	3.11e-06	YES	YES		
	5	rs17234657	2.42e-12	YES	YES	YES	18587394
	5	rs11747270	1.05e-06	YES	YES	YES	18587394
	7	rs7807268	5.43e-06	YES		YES	26192919
	10	rs10883371	5.23e-08	YES	YES	YES	21102463
	10	rs10761659	1.69e-06	YES	YES	YES	22936669
	16	rs2076756	7.55e-15	YES	YES	YES	21102463
**Hypertension (HT)**	1	rs10889923	1.38e-05				
	15	rs2398162	6.01e-06	YES			
**Rheumatoid arthritis (RA)**	1	rs6679677	<1.0e-15	YES	YES	YES	20453842
	4	rs3816587	7.28e-06	YES	YES		
	6	rs9272346	7.38e-14	YES	YES		
	22	rs743777	1.01e-06	YES		YES	23143596
**Type 1 diabetes (T1D)**	1	rs6679677	<1.0e-15	YES	YES	YES	19430480
	2	rs231726	1.43e-05			YES	30659077
	4	rs17388568	3.07e-06	YES	YES	YES	21829393
	6	rs9272346	<1.0e-15	YES	YES	YES	18978792
	12	rs17696736	1.56e-14	YES	YES	YES	18978792
	12	rs11171739	8.36e-11	YES		YES	19430480
	13	rs4769283	1.20e-05				
	16	rs12924729	7.86e-08	YES	YES	YES	17554260
**Type 2 diabetes (T2D)**	2	rs6718526	1.00e-05		YES	YES	20418489
	4	rs1481279	9.44e-06	YES	YES	YES	28869590
	6	rs9465871	3.38e-07	YES	YES	YES	21490949
	10	rs4506565	5.01e-12	YES	YES	YES	23300278
	12	rs1495377	7.21e-06	YES	YES	YES	22885922
	16	rs7193144	4.15e-08	YES	YES	YES	22693455

The DeepCOMBI method finds 39 significant associations. According to the fifth column of Table 2, 31 of these SNPs were also discovered by the traditional RPVT approach because they have *P*-values <10^–5^. The other 8 of those 39 SNPs have *P*-values >10^–5^ and were hence not determined to be associated with the disease with RPVT in the original WTCCC publication. They are of special interest because they represent additional SNP disease associations that the traditional analysis of the data was not able to identify. Out of these eight novel discoveries, six have been validated independently in later GWAS or meta-analyses: rs7570682 on chromosome 2 and rs1375144 on chromosome 2 for bipolar disorder; rs6907487 on chromosome 6 for coronary artery disease; rs12037606 on chromosome 1 for Crohn’s disease; rs231726 on chromosome 2 for type 1 diabetes and rs6718526 on chromosome 2 for type 2 diabetes.

On the other side, two out of the eight novel DeepCOMBI SNPs with *P*-values > 10^–5^ have not yet been replicated in any independent GWAS or meta-analyses. They have also not been identified by the COMBI method. Those entirely novel DeepCOMBI discoveries are rs10889923 on chromosome 1 for hypertension and rs4769283 on chromosome 13 for type 1 diabetes. To determine whether those two SNPs are biologically plausible discoveries for an association with the respective disease, their genomic regions were investigated in terms of functional indicators. Strong evidence of potential functional roles in the diseases was found.

First, rs10889923 maps on an intron for *NEGR1* (neuronal growth receptor 1), a very important gene many times linked to obesity, body mass index, triglycerides, cholesterol, etc. and many other phenotypes highly correlated with hypertension (66–68). Even though *NEGR1* has been associated with many phenotypes in the GWAS Catalog, no GWAS has yet been able to directly link it to hypertension. Furthermore, rs10889923 is part of a high LD region (according to LDmatrix Tool (69)) with variants that have been reported to be significantly associated with a number of psychiatric disorders and phenotypes, e.g. educational attainment (rs12136092 with *P*-value $ < 1{e^{ - 11}}$ and a degree of LD ${R^2} = 0.86$ to rs10889923; rs11576565 with *P*-value $ < 1{e^{ - 8}}$ and ${R^2} = 0.63$) (17). This link suggests a potential connection between hypertension and related phenotypes with mental traits. rs10889923 can thus altogether be considered an excellent candidate for association with hypertension.

Second, rs4769283 on Chr. 13 lies in an intergenic region very close to a gene called MIPEP (mitochondrial peptidase) that cannot be directly linked to T1D but is reported as a significant eQTL (expression quantitative trait locus) for two other genes, namely C1QTNF9B and PCOTH (70). Thus, MIPEP and therefore rs4769283 significantly control expression levels of mRNAs from these two genes in a particular tissue. Most remarkably, rs4769283 is a significant eQTL (with *P*-value $ = 1.1{e^{ - 6}}$) for C1QTNF9B (complement C1q and tumor necrosis factor-related protein 9B) in (among several other tissues) the pancreas, which produces very little or no insulin in T1D patients. So even though the association of rs4769283 with Type 1 diabetes is not an obvious one, it is indeed an interesting novel discovery of the DeepCOMBI method.

To present a more condensed view of these discoveries, Table 3 summarizes the findings of the three competitor methods, RPVT, COMBI and DeepCOMBI. When no screening step is conducted and RPVT *P*-values are calculated for all SNPs, 68 locations with *P* < 10^–5^ were identified as significant RPVT hits. COMBI and DeepCOMBI both apply a learning-based SNP preselection step and thus, find fewer significant associations. The DNN-based approach to this is seen to be more conservative than the SVM-based one, with only 39 identified locations of DeepCOMBI in comparison to 53 findings of the COMBI method. Even though the DeepCOMBI method finds fewer significant SNPs than COMBI, the number of independently replicated SNPs of DeepCOMBI (31 replicated SNPs, yielding a precision of 79%) is identical to that of COMBI (31, precision = 58%) and almost identical to that of RPVT (33, precision = 49%). In addition, the DeepCOMBI method misclassified only 8 of all unreplicated SNPs as associated with the disease (yielding an error rate of only 21%), while RPVT wrongly classified 35 SNPs (error rate = 51%) and the COMBI method made 22 mistakes (error rate = 42%). These observations are quantified with pairwise two-sided Fisher’s exact tests for the null hypothesis of equal error rates for both methods. They produce significant *P*-values for both the comparison of DeepCOMBI versus RPVT (Fisher’s exact test *P*-value of 0.002) and the comparison of DeepCOMBI versus COMBI (*P*-value = 0.0435).

**Table 3.** Quantitative summary of the significant findings of RPVT, COMBI and DeepCOMBI. For each of the three competitor methods, the numbers of replicated and unreplicated hits (i.e. the number of true and false positives) as well as precision and error rates are presented. Pairwise tests for the null hypothesis of identical distributions for DeepCOMBI and the two baseline methods are performed and the corresponding *P*-values are given

	Number of significant SNPs of		
	RPVT	DeepCOMBI method	COMBI method
**SNPs that have achieved *P* < 10^−^^5^ in at least one external study**	33 (49% precision)	31 (79% precision)	31 (58% precision)
**SNPs that have not achieved *P* < 10^−^^5^ in an external study**	35 (51% error rate)	8 (21% error rate)	22 (42% error rate)
**Overall**	68	39	53
**Pairwise *P*-value (two-sided Fisher’s exact test)**	DeepCOMBI versus RPVT = 0.002		DeepCOMBI versus COMBI = 0.0435

**Figure 7.**
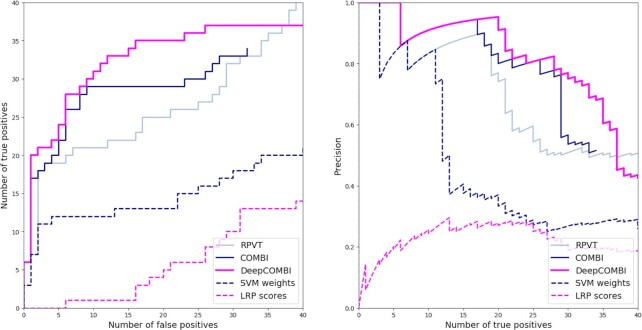
ROC and PR curves of DeepCOMBI and all competitor methods on WTCCC datasets. Performance curves of all methods averaged over all diseases and chromosomes are shown. ROC curves are presented on the left and PR curves on the right side. Replicability according to the GWAS catalog was used for validation.

Instead of investigating the significant findings of the three competitor methods achieved by matching a specific error level, it is also possible to examine the performance of those methods for different levels of error. By increasing the significance threshold of each method from very conservative ( ${t^*} = 0$, no significant SNPs) to very liberal ( ${t^*} = \infty$, all SNPs significant), we investigate here how the three methods perform under these circumstances. In Figure 7, we present the corresponding ROC and PR curves, where we interpret the replication of SNPs according to the GWAS catalog as a validation, i.e. we count a SNP as a true positive if it has achieved *P* < 10^–5^ in at least one external study. Overall, the findings obtained by the DeepCOMBI method are better replicated than those obtained by RPVT and COMBI for all levels of error. The performance metrics of the DeepCOMBI method are consistently better than that of RPVT and COMBI. The DeepCOMBI method finds more true positives for different levels of error and yields higher levels of precision for different levels of recall than COMBI and RPVT.

Figure 7 also shows the performance curves of the other two baseline methods that threshold SNPs solely based on raw LRP relevance scores or raw SVM weights, respectively. As we can view these two methods and RPVT as the individual components of the combinatorial approaches and neither of these three can achieve the same level of performance as COMBI and DeepCOMBI, it can be deduced that all subparts are essential. Only the combination of the two parts of the DeepCOMBI method (DNN with LRP explanation and statistical testing) can achieve the desired performance increase.

In an additional benchmark analysis, we compare the SNP discoveries of DeepCOMBI to those of Lippert *et al.* (21,27) who applied LMMs to the seven 2007 WTCCC datasets explicitly taking confounding factors such as population structure, family structure and relatedness into account and reported their results as a list of significant SNPs. For comparison, we access this list via Supplementary Table S2 presented by Lippert *et al.* (21) that includes 573 SNPs associated with six diseases (i.e. no reports for CAD). From this list, we exclude any SNPs that were discovered by them with a method other than LMM or that were not included in the dataset analyzed with the DeepCOMBI method (potentially due to different preprocessing steps) to ensure a fair comparison and avoid ascertainment bias. Since a lot of the remaining 110 SNPs are part of small SNP clusterings, we select representative markers for each locus through the LD pruning option in PLINK (63) and compute pairwise LD with a sliding window of two SNPs (with steps of 1 SNP at a time). We discard one SNP out of each pair if they are in high LD (*R*² ≥ 0.8). We run the final list of 72 SNPs, consisting of 1 discovery for BD, 0 for CAD, 19 for CD, 1 for HT, 3 for RA, 39 for T1D and 9 for T2D, through our validation pipeline using the same parameters that we use to evaluate the performance of DeepCOMBI (physical distance to tag-SNP: < 200 kb. LD with tag-SNP: *R*² ≥ 0.2). The corresponding results in comparison to the results of DeepCOMBI as presented in Table 2 are shown in Table 4.

**Table 4.** Quantitative comparison of the significant findings of the DeepCOMBI method and the univariate method from Lippert *et al.* (21,27). The numbers of discoveries and validated discoveries of the DeepCOMBI method from **Table 2** and the univariate analysis presented by Lippert *et al.* are presented.

	DeepCOMBI		Lippert *et al.* univariate analysis	
Disease	Discoveries	Validated discoveries	Discoveries	Validated discoveries
**BD**	4	4	1	0
**CAD**	4	2	0	0
**CD**	11	10	19	9
**HT**	2	0	1	0
**RA**	4	2	3	0
**T1D**	8	7	39	6
**T2D**	6	6	9	9
**Overall**	39	31	72	24
	*P*-value of two-sided Fisher’s exact test: < 0.0001			

Of the reported 72 SNPs, Lippert *et al.* (21) have discovered 24 true-positive SNPs that have been validated in external studies published after the WTCCC study, covering only three of the seven diseases (9 true positives for CD, 6 for T1D and 9 for T2D). The corresponding precision of 33% is much smaller than for DeepCOMBI, which achieved a precision of 79% by reporting 39 discoveries out of which 31 were validated. Not only does the DeepCOMBI method give rise to more validated discoveries in total and has higher precision than LMMs, but its discoveries also cover the whole range of WTCCC diseases while the validated findings of Lippert *et al.* (21) are limited to CD, T1D and T2D. Overall, the advantage of DeepCOMBI over the univariate analysis of Lippert *et al.* (21) is significant with a *P*-value $ < 0.0001$ (two-tailed Fisher’s exact test for a 2×2 contingency table with replicated versus unreplicated SNPs, i.e. 31 versus 8 for DeepCOMBI and 24 versus 48 for Lippert *et al.* (21)).

## CONCLUSIONS AND DISCUSSION

Numerous different approaches for the analysis of GWAS have been introduced since the first of its kind was published in 2002. Traditionally, they focus either on accurate phenotype prediction (1–5) or the identification of SNP phenotype associations (6–10). At first, most of these approaches were of a purely statistical nature (15,16), but since machine learning has become increasingly important in data science, it has also found its way to the investigation of genetics data. A large range of all kinds of machine learning-based tools have been proposed and investigated: regression and classification approaches, non-penalized and penalized methods, linear and nonlinear models (4,28–30,32,33). A number of very well-performing methods introduce the combination of traditional statistical testing concepts with more sophisticated machine learning tools (6,7,31). With the increasingly larger amounts of available data, deep learning-based approaches and artificial DNNs are now also being applied to GWAS datasets (41,42). However, most of these publications focus on pure classification or regression prediction tasks (28,43–45) rather than the identification of associated SNPs in the corresponding datasets (28,46).

To fill this gap and firmly based on the combinatorial approach of the COMBI method (31), the proposed DeepCOMBI method uses a deep learning-based phenotype prediction in combination with statistical testing for the identification of SNPs that are associated with the phenotype under investigation. DeepCOMBI could be considered an extension of COMBI, replacing the rather simple prediction tool of a linear SVM with a more sophisticated deep learning method and using the recent concept of explainability to uncover the decision-making process of DNNs and extract SNP relevance scores via LRP (48–50). To our knowledge, Romagnoni *et al.* (28) were the first and only scientists to use explainable AI in the context of GWAS and proposed to apply PFI. Even though they were able to identify some novel predictors, the prediction performance of their DNN was not better than that of traditional machine learning-based tools. In addition, PFI is a generalized, model-agnostic approach and more sophisticated methods specifically tailored to DNNs are available. Hence, DeepCOMBI makes use of deep Taylor-based explanation techniques by adopting LRP for the analysis of such GWAS data.

DeepCOMBI was shown to compare favorably to its main competitor COMBI on both generated controlled datasets as well as seven real-world GWAS datasets. These findings are in accordance with Romagnoni *et al.* (28), who found that deep learning-based methods can provide novel insights into the genetic architecture of specific traits. By applying LRP, we were able to leverage the power of DNNs and generate relevance scores that are less noise inflicted than the SVM importance scores of COMBI. In return, the preselection of candidates SNPs is better than that of COMBI, and a higher true positive rate and precision can be achieved for all levels of error. Since the COMBI method itself was shown before to outperform other combinatorial machine learning-based approaches (6,58,59) and a number of purely statistical analysis tools (21,27), it can be directly deduced that DeepCOMBI also outperforms those approaches. For example, Wasserman and Roeder (59) lose a great amount of statistical power by splitting the GWAS data under investigation into two parts, performing SNP preselection on one part and statistical testing on the other. This approach is significantly less successful in identifying SNP disease associations than COMBI and hence DeepCOMBI, who both perform all substeps on the complete (and therefore statistically more powerful) dataset. Another exemplary statistical method that was shown to be outperformed by COMBI and DeepCOMBI is based on linear mixed models (LMMs) proposed by Lippert *et al.* (21,27). Even though they test for pairwise epistatic interactions in addition to the univariate tests and address the issue of population stratification in GWAS, they still test genetic locations and pairs thereof individually instead of simultaneously. In comparison to COMBI and DeepCOMBI, which examine the genomic dataset as a whole, LMMs cannot achieve the same level of power and detection rates.

In addition to the main competitor method, COMBI, we also compared DeepCOMBI to the baseline methods of RPVT, raw LRP relevance scores and raw SVM importance scores and showed that only the combination of deep learning and multiple testing show the desired performance increase, which cannot be achieved individually by one of these components.

A drawback of DeepCOMBI to consider might be that dense DNNs scale poorly with the number of SNPs studied. However, we have shown that DeepCOMBI performs well in combination with a *P*-value-based SNP preselection step.

Let us also address potential issues arising from the optional feature selection procedure excluding SNPs with a *P*-value smaller than the threshold κ from the analysis. Most importantly, it is unlikely that this procedure will cause our method to be overly conservative by excluding promising candidate SNPs from the analysis since only SNPs with relatively low *P*-values will reach significance via the permutation test procedure in the final step of the method. However, one advantage of the DeepCOMBI method lies in automatically taking into account correlation structures and interactions between SNPs which might not show significant effects when examined individually. Therefore, the preprocessing step might dilute such effects and has to be applied carefully and only if necessary. A removed low-impact SNPs might not have reached significance itself if included but could have caused another more high-impact SNP to reach significance. On the other hand, let us point out that selecting a subset of features for DNN training might cause our method to introduce an optimistic bias and produce an increased amount of false positives (71,72). When the *same* dataset is used to select features *and* train a classifier, so-called feature selection bias can indeed become an issue (71,73). The cause of this bias is that the feature selection procedure is part of the training, but not external to the test samples (74). It is therefore essential to state that the current results might overestimate the real predictive effects of certain SNPs. However, we have evaluated our findings in an independent validation procedure with external GWAS studies which would inherently reveal a problem with selection bias if existent. However, in spite of any potential feature selection bias, we have shown that, in combination with the applied feature selection method, DeepCOMBI objectively performs better than methods without feature selection. It achieves higher power and precision than the examined competitor methods while yielding fewer false and more true discoveries when its results are validated on later GWAS studies. Nevertheless, introducing a more sophisticated feature selection procedure could be the focus of future research endeavors. Inspiration can be taken from a number of previous publications which focus on addressing related issues in the field of biomedical research (72,73,75,76).

Furthermore, the DeepCOMBI method does not account for confounding factors such as relatedness which is studied as one of the most important challenges in statistical genetics (77,78) and GWAS typically involve related individuals. We, therefore, dedicate the following sections to address potential issues with confounding factors and selection biases caused—among other things—by including related individuals, i.e. non-independent and identically distributed (non-i.i.d.) samples in the datasets under investigation. In general, machine learning and, more specifically, neural networks are formally based on the assumption of i.i.d. random variables (79,80). This assumption enables the simple development of efficient theories (81) and methods (53), causing most ML-based research to focus on learning with i.i.d. data. In particular, neural networks require independent samples to avoid getting stuck in the training phase, finding local optima or overfitting and not generalizing well to external data (79,80). When one datapoint influences and is hence not independent of another datapoint, this violation of the i.i.d. assumption is called an interdependency (80). In practice, non-i.i.d. samples are often assumed to be only a theoretical issue and included in analyses nonetheless (82). In part, that is because they can be accounted for by, for example, choosing the architecture and hyperparameters of the network appropriately (80). On the other hand, in some areas, related samples seem to actually have no effect on the overall performance of a neural network. This is particularly prevalent in the field of image recognition, where datasets often include related images which are rarely addressed as such explicitly (37). With large enough datasets, where small clusters of dependent samples (close or distant relatives in our case) are selected at random from the entire data distribution (i.e. population), the effects are often negligible. However, selection bias due to related samples can indeed become an issue when the complete dataset is too small. In addition, interdependencies are very common in computational biology since the corresponding datasets are often composed of related organisms (27), phenotypes (83) or features (84). Since ignoring these confounding factors in the corresponding datasets can indeed cause false-positive discoveries that cannot be replicated on independent data (85), a number of approaches have been developed to account for population structures due to relatedness between samples (21,27,78,80,86,87). Some of these approaches extend existing algorithms to account for non-i.i.d. data (82,88). Others have been proposed to address time-structured dependencies (89). For example, recurrent neural networks are used, in contrast to feedforward networks, to model interdependent data by allowing connections between a neuron and neurons of the same or previous layers (90). However, this architecture is designed to account for non-i.i.d. data caused by sequentially dependent data (e.g. time series data) and not for dependencies arising from grouped relatedness as found in GWAS. Considering this kind of non-i.i.d. data, a number of methods have been presented to correct for population structures following both statistical (27) as well as ML-based approaches (86). Recently, very large datasets containing related samples have been investigated (91–93) to examine the effect of interdependencies in GWAS using LMMs as one of the most widely used tools to account for relatedness regressing the phenotype measures on a relationship matrix of fixed effects. LMMs capture confounding factors such as population structures, family structures and relatedness simultaneously, without the exact knowledge of which are present and without the need to tease them apart (27). The underlying idea is to model the output based on a mix of factors: the functional ones we are trying to identify and the fixed ones that just arise from confounding factors. To evaluate the effect of interdependencies and population structures in the WTCCC datasets under investigation here, we compared the performance of DeepCOMBI to that of the LMM approach proposed by Lippert *et al.* (21,27) in a benchmark analysis and found that the DeepCOMBI method yields both more discoveries that were validated in external studies and higher precision than the LMMs as presented by Lippert *et al.* (21,27). The validated discoveries of DeepCOMBI are also more broad covering the whole range of diseases while the validated findings of Lippert *et al.* (21) are limited to CD, T1D and T2D. In general, the comparison of the two methods is favorable to DeepCOMBI with a significant *P*-value of $ < 0.0001$ (two-tailed Fisher’s exact test). Even though the comparison of our method with an approach correcting for population structures was highly favorable to DeepCOMBI, it is not possible to conclude that relatedness within the 2007 WTCCC dataset is limited and non-i.i.d. samples have no impact at all. However, the unique benefit of our method was demonstrated when we revealed biological signals with state-of-the-art methods in datasets of almost 15 years of age that standard GWAS analyses have not been able to capture in any dataset published to this day. Furthermore, we clearly demonstrate the replicability of our findings, which is a robust way (94) and the gold standard (95) of verifying the biological signals underneath the investigated data.

In conclusion, DeepCOMBI, a novel AI-based method, was proposed for the analysis of GWAS data. After training a carefully designed DNN for the classification of subjects into their respective phenotype, the concept of explainable AI is applied by backpropagating the class prediction score to the input layer through the network via LRP. The resulting SNP relevance scores are used to select the most relevant SNPs for multiple testing in combination with a permutation-based thresholding procedure. On both generated, controlled datasets as well as seven real GWAS datasets, DeepCOMBI was shown to perform better than a number of competitor methods in terms of classification accuracy of the DNN and in terms of ROC and PR curves when using either the generated labels or replicability in external studies as a validation criterion. In addition, two very promising, entirely novel SNP disease associations were discovered. Located on an intron for *NEGR1*, an important gene many times linked to obesity, body mass index and other correlated factors, rs10889923 on chromosome 1 was found to be significantly linked to hypertension. Another novel location found by DeepCOMBI to be associated with type 1 diabetes is rs4769283. It is part of an intergenic region on chromosome 13 and was previously found to be an eQTL for C1QTNF9B in the pancreas, the affected organ in T1D patients.

Future work on the subject of deep learning and explainable AI in the context of analyzing GWAS datasets could focus on one of the three steps of DeepCOMBI. In the first step, DNNs with different architectures or other suitable analysis tools could be investigated. For example, future research could aim to harness the potential of convolutional networks (96) in this application. By integrating multiple output nodes for multiple phenotypes, the DNN could also be extended to cover multivariate output variables and examine multimorbidities. DNNs can easily be adjusted to nonbinary phenotypes. Improvement ideas for the second step of the proposed method include the application of different explanation methods (e.g. PFI) or LRP backpropagation rules, for example, according to the layer types, as advised by Montavan *et al.* (48). Great potential lies in finding more sophisticated ways to combine the local LRP explanations of each individual subject to a single global explanation used for SNP selection. A very promising candidate would be a method called SpRAy (97), which clusters the individual explanations and simplifies the identification of explanatory structures in subsets of subjects. Future research work considering the third step of DeepCOMBI might examine the effects of replacing the ${\chi ^2}$test with a different, more sophisticated kind of test, e.g. investigating pairwise hypotheses or other multivariate effects.

In addition, fully evaluating our approach on datasets with related samples and integrating DeepCOMBI with LMMs or adapting it to correct for relatedness could be the focus of future research. A dataset much larger with respect to both samples and SNPs would have to be investigated. For example, the UK Biobank dataset could be the focus of such an analysis since it includes about 500 000 subjects for whom hundreds of phenotypes have been registered (98). Some of the subjects in this dataset are related and it has been the testing ground for several algorithms to account for relatedness (91–93). To elaborate on potential ways interdependencies could be accounted for in extensions of the DeepCOMBI method, let us first comment on Xiong *et al.* (88) who have shown that in certain settings performances of neural networks can be improved when they are explicitly designed to handle non-i.i.d. samples. They propose to adopt the concept of mixed effect models (99,100) in classical statistics to convolutional DNNs for gaze estimation with multiple measurements from the same individuals and achieve a 10–20% increase in performance. A similar approach could be adapted for the DeepCOMBI method where the interdependencies come from related subjects instead of repeated measurements. In this context, LRP, as applied in DeepCOMBI, has been successfully applied to convolutional DNNs for example by Harley (101). A combination of the two approaches could be developed to adapt our approach to account for related subjects by employing mixed effect models in convolutional DNNs and applying LRP explanation to identify associated SNPs. Another potential focus of future research including the proposed method could lie in identifying and quantifying the effect of small groups of non-i.i.d. samples (i.e. families) in GWAS datasets. This could be achieved by clustering the LRP explanations of all samples as proposed by Lapuschkin *et al.* (97) and identifying the locations of related individuals in the resulting clustering structures. If the subjects of a family end up in the same clusters in such an analysis, the effect of the underlying selection bias could be visualized and it could be shown that SNPs that have an effect in a specific family are not necessarily associated with the trait in the general population.

While the focus of this work is on SNP discovery, let us address how its results relate to risk prediction and in which ways the DeepCOMBI method might be used to estimate heritability. There are several state-of-the-art tools and approaches that can estimate polygenic risk scores for diseases using SNPs from classic GWAS (102). They focus on examining how well a certain set of SNPs separates cases from controls and, in the last instance, identifying the contribution of a specific SNP to the heritability of a specific disease. Future research on the DeepCOMBI approach could potentially address these questions and use its results, a ranked list of SNPs, for risk prediction and heritability estimates. In this context, let us point out that the DeepCOMBI method is composed of a class prediction step with DNNs and a subsequent identification step of the SNPs that increase the risk of a high prediction score. Predicting the risk of new subjects can be achieved by providing their genetic sequence as input to the trained DNN and interpreting the prediction score as a risk score. The method inherently contains a measure of the overall contribution of the genetic component to the disease, namely the explanation scores learned from LRP, which—per definition—sum up to the prediction score of each sample. Running LRP as a follow-up investigation after calculating prediction scores for new subjects will highlight the SNPs that had the largest effect on this specific prediction. Averaging these scores for multiple subjects provides an estimate of the general contribution of a specific SNP to the disease under investigation. Beyond the intrinsic risk prediction properties of DeepCOMBI, let us discuss some potential downstream directions for estimating heritability. Many DNN-based approaches have been proposed for post-GWAS prioritization (103–105), but very few are designed for SNP discovery. To our knowledge, applying DNN-based methods to calculate risk predictions or heritability estimates could open up a new field of research that has not been given much attention. Given that DeepCOMBI is one of the first tools designed to use DNNs in GWAS for SNP discovery, and not for post-GWAS prioritization, its results could be used for heritability partitioning, where a particular set of SNPs is evaluated for enrichment in explaining the percentage of heritability of a specific phenotype. A widely used approach addressing this issue is the LD-score (LDSC) (106). An adoption of heritability partitioning through LDSC to extend our deep learning-based approach could be the subject of a specialized research project on heritability and risk estimates for patients in a larger cohort.

Besides the discussed objective of GWAS to extrapolate risk predictions, our study could also be used to increase the statistical power of limited sample-size GWAS studies, offering an easy-to-use tool available to situations of limited resources or rare phenotypes where it is not possible to gather large sample sizes. Individual GWAS loci have already shown the potential for large-scale prioritization by providing novel biological insights and potential drug targets and drug repositioning opportunities (107).

## DATA AVAILABILITY

DeepCOMBI source code is available at https://github.com/AlexandreRozier/DeepCombi.

The 2007 WTCCC dataset (14) analyzed during the current study is not publicly available. Access can be requested from the owners at https://www.wtccc.org.uk/info/access_to_data_samples.html and https://www.sanger.ac.uk/legal/DAA/MasterController.

The code for simulating GWAS datasets is available at https://github.com/AlexandreRozier/DeepCombi.

## Notes

Present address: Juan Antonio Rodriguez, Center for Evolutionary Hologenomics, GLOBE Institute, University of Copenhagen, Copenhagen, DK-1353, Denmark.
